# Identification of the Framingham Risk Score by an Entropy-Based Rule Model for Cardiovascular Disease

**DOI:** 10.3390/e22121406

**Published:** 2020-12-13

**Authors:** You-Shyang Chen, Ching-Hsue Cheng, Su-Fen Chen, Jhe-You Jhuang

**Affiliations:** 1 Department of Information Management, Hwa Hsia University of Technology, New Taipei City 235, Taiwan; 2 Department of Information Management, National Yunlin University of Science and Technology, Douliou, Yunlin 64002, Taiwan; chcheng@yuntech.edu.tw (C.-H.C.); ananboy5566@gmail.com (J.-Y.J.); 3 National Museum of Marine Science & Technology, Keelung City 202010, Taiwan

**Keywords:** applications of medicine, cardiovascular disease, Framingham risk score (FRS), Framingham risk attributes, entropy-based rule model, machine learning techniques

## Abstract

Since 2001, cardiovascular disease (CVD) has had the second-highest mortality rate, about 15,700 people per year, in Taiwan. It has thus imposed a substantial burden on medical resources. This study was triggered by the following three factors. First, the CVD problem reflects an urgent issue. A high priority has been placed on long-term therapy and prevention to reduce the wastage of medical resources, particularly in developed countries. Second, from the perspective of preventive medicine, popular data-mining methods have been well learned and studied, with excellent performance in medical fields. Thus, identification of the risk factors of CVD using these popular techniques is a prime concern. Third, the Framingham risk score is a core indicator that can be used to establish an effective prediction model to accurately diagnose CVD. Thus, this study proposes an integrated predictive model to organize five notable classifiers: the rough set (RS), decision tree (DT), random forest (RF), multilayer perceptron (MLP), and support vector machine (SVM), with a novel use of the Framingham risk score for attribute selection (i.e., F-attributes first identified in this study) to determine the key features for identifying CVD. Verification experiments were conducted with three evaluation criteria—accuracy, sensitivity, and specificity—based on 1190 instances of a CVD dataset available from a Taiwan teaching hospital and 2019 examples from a public Framingham dataset. Given the empirical results, the SVM showed the best performance in terms of accuracy (99.67%), sensitivity (99.93%), and specificity (99.71%) in all F-attributes in the CVD dataset compared to the other listed classifiers. The RS showed the highest performance in terms of accuracy (85.11%), sensitivity (86.06%), and specificity (85.19%) in most of the F-attributes in the Framingham dataset. The above study results support novel evidence that no classifier or model is suitable for all practical datasets of medical applications. Thus, identifying an appropriate classifier to address specific medical data is important. Significantly, this study is novel in its calculation and identification of the use of key Framingham risk attributes integrated with the DT technique to produce entropy-based decision rules of knowledge sets, which has not been undertaken in previous research. This study conclusively yielded meaningful entropy-based knowledgeable rules in tree structures and contributed to the differentiation of classifiers from the two datasets with three useful research findings and three helpful management implications for subsequent medical research. In particular, these rules provide reasonable solutions to simplify processes of preventive medicine by standardizing the formats and codes used in medical data to address CVD problems. The specificity of these rules is thus significant compared to those of past research.

## 1. Introduction

This section explores the research background and problem in the relevant medical domains, the research gaps and motivations, and the study goals and research objectives.

### 1.1. Research Background and Research Problem

Cardiovascular disease (CVD) is one of the main causes of death [1] in most countries and likely results in related problems in the blood vessels or the heart, such as cerebrovascular disease (i.e., stroke), congenital heart disease, coronary heart disease (CHD) [2], heart failure, peripheral artery disease, raised blood pressure (i.e., hypertension), and rheumatic heart disease. It is estimated that 25 million new cases of heart disease each year are diagnosed. Thus, CVD has been a key cause of death by a serious illness in recent years [1,3]. Effective identification of the risk factors of CVD is therefore highly important for clinical research for long-term therapy and prevention to reduce wastage of medical resources. Based on past studies, the main risk factors of CVD include an unhealthy diet, harmful use of alcohol and tobacco, and physical inactivity. According to a report from the World Health Organization [4], in 2015, there were 17.7 million deaths related to CVD, representing 31% of global deaths and a higher mortality risk than that of general diseases. Among these deaths, 6.7 million died from a stroke, and 7.4 million died from CHD. In 2015, 82% of premature deaths (under age 70) were caused by non-communicable diseases (NCDs) in low- and middle-income countries (LMICs), 37% of which resulted from CVD. In recent decades, the mortality rate of CVD has declined in high-income countries (HICs). Conversely, it has increased in LMICs surprisingly rapidly. However, it is possible that efforts to improve healthcare interventions against risk factors of CVD result in a significant reduction of deaths and medical resources and socioeconomic burdens. Thus, identification of the CVD problem is first emphasized in this study. This problem poses significant challenges and an opportunity in advanced preventive medicine for medical application research.

Given the above characterizations, it is clear that the issue of identifying CVD is urgent. Thus, the related issue of the CVD problem is a key research concern and is one of the core research goals of this study. The benefits of this study are an exploration of the risk factors of CVD fatal diseases and effective methods for their identification. In the context of past studies, these are novel findings.

### 1.2. Research Gaps and Motivations

Table 1 lists the top five causes of death in 2015 in Taiwan, as reported by the Ministry of Health and Welfare [5]. It is clear that CVD has been the second most common cause of death since 2001. CVD not only results in higher medical resource expenses but also greater long-term expenditure for countries and individual families. To tackle this severe problem, developing countries have dedicated funds to CVD prevention and treatment through national education and training resources, thereby reducing incidence and mortality rates [6]. However, due to changes in lifestyle and eating habits in recent decades, incidence rates of CVD among younger people have experienced a growing trend. Thus, early detection and prevention of CVD and closing this research gap related to younger people have become critical issues. From the perspective of preventive medicine, the design of an effective predictive (classification) model to help doctors in the early and accurate diagnosis of CVD is required, and the effectiveness and quality of prevention and treatment need to be improved. Thus, this study is motivated by the following. First, the classification function of machine learning techniques for mining meaningful CVD rules (knowledge) from large amounts of data is a valuable approach. Notably, classification techniques have been successfully applied in medical fields, such as breast cancer [7,8] and heart diseases [9,10], by studying past research. Furthermore, Boursalie et al. [11] efficiently utilized a support vector machine (SVM) classifier to monitor CVD via effective features and characteristics. Second, it is clear from a limited literature review that using effective risk factors (e.g., Framingham risk attributes) to mine CVD data is efficient and effective [1]. Although past studies have used features of Framingham risk to help prevent CVD [12], they have rarely used a hybrid model to integrate the Framingham risk score and classification techniques to address the issue of CVD for doctors and patients. Finally, the Framingham risk score is an effective instrument that can be used to build a hybrid prediction model to accurately diagnose CVD. Thus, this study is beneficial both theoretically and practically.

Based on the above descriptions, serious diseases (e.g., CVD) require greater healthcare and the identification of potential preventable materials or approaches at both the country and individual family levels. The challenge related to CVD has thus attracted significant attention from practitioners and academics. Therefore, the study examines the noteworthy and important issue of effectively identifying the risk factors of CVD.

### 1.3. Study Goals and Research Objective

The CVD problem reflects an urgent issue. A high priority has been placed on long-term therapy and prevention to reduce the wastage of medical resources via the greater use of preventive medicine, particularly for developed countries. Furthermore, popular data-mining methods have been well learned and studied, with excellent performance in medical fields. Thus, identification of the risk factors of CVD using popular data-mining techniques is also a major goal of this study. In addition to addressing the issue of CVD, the study also aims to reduce the wastage of medical resources and financial expenditure.

In data-mining fields, various emerging machine learning models have been found to be superior in their application to healthcare issues [13]. Thus, in this study, a hybrid predictive model was first built to identify CVD using five well-performing classifiers in the medical domain, namely, decision tree (DT), multilayer perceptron (MLP), random forest (RF), rough set theory (RST), and SVM, and simultaneously measure the performance of the Framingham risk score with full attributes. In practice, the study used two real datasets collected from a Taiwan teaching hospital with CVD cases and a public Framingham dataset from the Internet for the further benefit of preventive treatment. To summarize, this study has three research purposes: (1) construction of a hybrid predictive model of CVD based on diverse classifiers and the Framingham risk score; (2) identification of the key determinants of CVD by attribute selection methods and extraction of comprehensible entropy-based rule sets based on DT; and (3) provision of analytical results and research findings with management implications to healthcare providers, physicians, and patients as a useful medical reference. The entropy-based method is beneficial for information gain [14].

The remainder of this paper is organized as follows: A literature review of the study issues, including CVD, the Framingham risk score, and the five classification techniques, is provided in Section 2. The research concepts justifying the procedure of the proposed method are presented in Section 3. The analysis results and the core research findings from the experiments are provided in Section 4. Finally, Section 5 concludes and suggests subsequent research.

## 2. Related Work

The literature and concepts related to the identification of CVD, the Framingham risk score, and the five classification techniques are introduced and explored in this section.

### 2.1. Cardiovascular Disease and Its Identification Applications

CVD is a disease related to the blood vessels and heart, and coronary artery diseases (CAD, e.g., heart attacks or angina). Its major clinical symptoms have various levels of severity and include cardiomyopathy, congenital heart disease, hypertensive heart disease, peripheral artery disease, rheumatic heart disease, and stroke [15]. In clinical practice, CVD and cerebrovascular disease have a cause-and-effect relationship [16]; thus, CVD is a sign of a serious illness because CVD patients usually also have various extensions of serious chronic diseases (e.g., hypertension, stenocardia, hyperlipidemia, hyperuricemia, diabetes mellitus, and obesity). Similarly, other heart diseases, such as coronary sclerosis, valvar heart disease, and ventricular fibrillation arrhythmia, and serious conditions, such as cerebral embolism and myocardial infarction, are complicated and exacerbated by the negative effect of CVD. Henriksson et al. [17] provided evidence that cardiorespiratory fitness, body mass index, and muscular strength in adolescents can lead to later chronic incapacity and CVD disability, including cerebrovascular disease, heart failure, and ischemic heart disease.

The risk factors of CVD must first be explored and identified. These risk factors are classified into two categories. The main risk factors include diabetes mellitus, an older age, family history, hyperlipidemia, hypertension, and obesity. Subordinate risk factors include high uric acid, narrow heart disease, myocardial infarction, arrhythmia, cardiogenic shock, peripheral vein disease, and smoking. The three main risk factors of CVD disease are as follows:

(1) Hypertension: Blood pressure refers to the arterial blood pressure based on the vessel wall and is divided into two types: diastolic blood pressure (DBP) and systolic blood pressure (SBP). According to a WHO standard, normal SBP is between 90 and 140 mmHg, and DBP is between 50 and 90 mmHg. With hypertension, the value of SBP/DBP exceeds 140/90 mmHg. Based on a report from the USA entitled “Hypertension prevention, detection, and treatment criteria”, SBP has an average of 140 mmHg, but DBP is over 90 mmHg [18]. In particular, McManus et al. [19] showed by a meta-analysis that the primary prevention of CVD consisted of treating patients with blood pressure under 140/90 mmHg. Thus, hypertension is a risk factor for CVD, and it is important to lower blood pressure.
(2) Hyperlipidemia: Hyperlipidemia refers to a higher-than-normal concentration of cholesterol or triglycerides in the blood [20]. Dyslipidemia for non-normal hyperlipidemia is the major cause of atherosclerosis with high cholesterol, high triglycerides, and their combination, which may increase the probability of coronary and heart diseases, and results in CVD. Moreover, Burkhardt [20] indicated that elevated plasma levels of low-density lipoprotein (LDL) are modifiable major risk factors for atherosclerotic CVD. However, high-density lipoprotein (HDL) is a good factor for identifying CVD. Thus, subsequent researchers should identify and measure crucial factors causing LDL and HDL in Taiwan’s CVD patients.
(3) Tobacco: Tobacco use multiplies the risk of CVD through a number of negative mechanisms [21], such as damaging the endothelium of the blood vessels, increasing fatty deposits of arteries, increasing clotting, raising LDL-cholesterol (LDL-C) and lowering HDL-cholesterol (HDL-C), and promoting coronary artery spasms. Banks et al. [21] noted that tobacco use is a major source of CVD mortality and morbidity. In particular, nicotine, an addictive component of tobacco, raises blood pressure and accelerates the heart rate. According to a report from the World Heart Federation [22], if one started smoking as a child, the potential risk of suffering CVD is many times higher compared to those who began as a legal adult. Furthermore, passive smoking is harmful and increases the risk of CVD; chewing tobacco and taking snuff are also dangerous.

In addition to these three main risk factors, other potential factors need to be further examined and identified effectively to enable the results to be compared to those of the literature.

### 2.2. Framingham Risk Score

Identifying and preventing the major modifiable risk factors of CVD in advance can lower the prevalence rate for related heart disorders [1]. In 1948, the Framingham Heart Study (FHS), directed by the National Heart Institute, USA, began studying cardiac disease (heart disease) for health research purposes. At that time, the general cause–effect relationship between heart disease and stroke was not known. Subsequently, however, the mortality of CVD has steadily increased, reaching epidemic levels in the USA at the beginning of the current century [23]. The main aims of this project were to identify the common characteristics and risk factors of CVD patients by monitoring participants who had not yet suffered strokes and heart attacks due to CVD. The FHS investigated the risk factors of CVD in well-designed experimental groups, which consisted of 5209 people from Framingham, Massachusetts, USA, between the ages of 30–62 and free of CVD [23].

Investigators of the FHS first developed the risk equation of CHD [24] for clinicians to predict coronary disease from 1950 to the mid-1960s, reflecting early efforts from investigators. The long-term studies of the FHS found data with respect to related heart diseases. In addition to age and gender, the risk factors of CVD were found to include high SBP, serum cholesterol, glucose intolerance (e.g., diabetes), and left ventricular hypertrophy (LVH); furthermore, the FHS further identified lifestyle factors, such as sedentary lifestyle, eating an unhealthy diet, and cigarette smoking. In addition, the influence of HDL-C [25] was measured and is reflected in the equations. Measurement of total HDL-C was also found to be superior to that of serum cholesterol as a predictor of CVD. Based on the above, high blood cholesterol, high blood pressure, diabetes, obesity, physical inactivity, and smoking were defined as the main risk factors of CVD. Other characteristics, such as age, blood triglycerides, gender, HDL-C level, and psychosocial issues, have also been determined as relevant factors over the years [26]. More important, behavioral mechanisms for lowering the main risk factors of CVD showed a positive relationship with modern medical trends, which resulted in an emerging treatment and prevention strategy for the practice of clinical medicine.

The National Heart, Lung, and Blood Institute (NHLBI) and Framingham investigators continue to research genetic factors (genetic material or genetic patterns) of CVD using new diagnostic technologies. Hojat et al. [1] compared the risk factors of CVD among male and female nurses and found that men have a higher risk and more complications of CVD than women. According to the FHS conversion standard, the index factors of Framingham risk are age, blood pressure, diabetes status (e.g., fasting glucose), gender, HDL-C level, smoking status, and total cholesterol level [27,28]. Consequently, the transformed scores of the above factors are listed in Table 2, Table 3 and Table 4 below.

### 2.3. Classification Technology

This subsection uses advanced classification machine learning technology to highlight the purpose of dealing with large amounts of medical data from clinical databases. This study applied five noted classification technologies, namely, RST, DT, RF, MLP, and SVM, which were selected because they were previously used in medical classification and prediction of healthcare problems and found to have superior performance.

#### 2.3.1. Rough Set Theory and Its Practical Applications

RST was developed initially by Pawlak [29]. It classifies by analyzing incomplete, unspecific, or imprecise information and extracts decisional rule-sets. In the data analytics community, RST is used for non-statistical techniques. Its essential conception is to use β-lower and β-upper approximations for a given set, and the β-lower and β-upper approximations form formal classification information in a specific domain. Objects shape a part of a subset, and objects that possibly shape a part of a subset characterize the subset of the β-upper approximations and the subset generated by the β-lower approximations. Each subset determined by β-lower and β-upper approximations is called a rough set [30,31,32]. RST is widely used today in medical research, process control, and database attribute analyses. Other fields include heart disease [33], medical data diagnosis [34], multiobjective attribute reduction [35], the safe use of medical devices [36], short text data [37], textile applications [38], and the supply chain of auto spare parts [39].

RST provides four benefits when processing classification problems: (1) It is unnecessary to use external information; (2) it is appropriate for studying qualitative and quantitative data; (3) hidden information from given data is discovered through decisional rule-sets and is thus easier to interpret; and (4) extracted decisional rules eliminate redundant or useless information from the given dataset. In further exploration, RST is implemented in six steps [40]:

(1) Defining the problem faced: The first stage in RST is to define the problem to be faced by explaining and forming the decisional attributes by means of condition attributes and creating decisional rule-set units for future use following the function of classification parts.
(2) Preprocessing the data collected: In this step, data values collected from the units of both the condition attributes and decision attributes are preprocessed. RST allows these attributes to represent types of categorical data; therefore, if there are continuous data in the condition attributes of a given dataset, they can be first discretized into a meaningful form by applying various transforming algorithms [41].
(3) Forming the information and decision systems: The decisional information system (DIS) is framed in accordance with the data collected. A DIS is a table that defines the units of condition attributes, and this table is built with the decision attribute in the last column.
(4) Defining the indiscernibility relations and setting approximations: First, the data used to address the decisional problems occur in a DIS by determining the relationships of indiscernibility. However, the derived system may encounter larger scales of tables due to excessive attributes or unnecessary attributes that repeat similar parts, which has a negative impact on the classification performance of the model. Thus, this negative effect should be reduced as much as possible against the given data of attributes. Second, defining the condition attributes and the decision attributes with the current properties to a certain class after using the relationships of indiscernibility should not be specific for determining these set approximations. In this case, the unit that completely belongs to a given set or the unit that possibly belongs to that set is defined by the functions of the β-upper and β-lower approximations of spaces.
(5) Defining the sets reduced: Attributes in a DIS are filtered from the table without losing their fundamental properties. We assume that one attribute ***a*** belongs to the ***B*** set, where ***B*** ⊆ ***A***. For the ***B*** set, if *I*(***B***) = *I*(***B*** − {***a***}), then attribute ***a*** is unnecessary; however, for a specific case in which the above formula is unfulfilled, attribute ***a*** is necessary from the ***B*** set [42]. The unnecessary attributes are removed from the subsets, and these are then called the reduced sets [43].
(6) Forming decisional rules: Decisional rules are formed and produced by accounting for the attributes of reduced sets [44], and their propositions highly depend on the values of the condition attributes against the decision attribute. Importantly, each decisional rule-set has an accuracy percentage, and the total accuracy rate can then be acquired.

The literature suggests that RST is suitable for use in medical fields [45], and it was thus selected for this study.

#### 2.3.2. Decision Tree and Its Real Applications

DT is a regression or classification approach for forming a tree-based rule structure from a supervised algorithm. DT uses supervised learning technology and has a classification (or prediction) function for well-known machine learning techniques in various application domains. Thus, DT results in fast formations and produces an entropy-based decision rule of easy if–then–else interpretations in a variety of fields; it has become a common application technique among classification approaches [46,47]. DT algorithms contain two stages: developing a tree and pruning a tree. For the first phase, DT selects a better suitable attribute from a split training dataset. The final outcome only applies to that data, and the common training splits of the sub-dataset target a specific class. The repetition or recursion functions concerning the selection attribute and splitting-set construct internal nodes and a root node. In the pruning-tree phase, some data from the trained sub-dataset may have an inappropriate branch after the DT structure is built, i.e., overfitting, and these inappropriate branches must be eliminated to improve the classification performance [48,49].

In the DT community field, a core algorithm for employing a top-down structure is called iterative dichotomizer 3 (ID3) [50]. ID3 uses information theory to build an entropy-based tree rule for information gain and entropy. The key concept of quantifying information is called entropy and is mainly used for calculating the homogeneity of the given data by the ID3 algorithm. The entropy will be zero if the data sample is homogeneous; conversely, it will be one if the data sample is completely heterogeneous. Information gain is used for training a decisional tree and is based on the reduction in entropy when a given dataset is converted. A calculation is made by comparing the entropy of this dataset before and after conversation; thus, the highest information gain will be used to construct a decisional tree in the process of finding attributes. The entropy is defined as the following Equation (1) by using a frequency table of one variable:

Entropy (*E*) = − (*p* × log(*p*) + (1 − *p*) × log(1 − *p*)), (1)

where *p* is the probability of one class.

A collection set *S* of outcomes is determined, and the entropy is formatted as:

(2) $$E\left(S\right)=\sum_{i=1}^{n}(-p_{i}\log_{2}p_{i}),$$

where $p_{i}$ is the proportion of *S*, which belongs to class *i*?

Gain (*S*, *A*) is the information gain of a case set *S* on attribute *A* and is formatted as:

(3) $$Gain\left(S,A\right)=E\left(S\right)-\sum \frac{\left|S_{v}\right|}{\left|S\right|}E\left(S\right),$$

where *v* is a value of *A*, $S_{v}$ is a subset of *S*, $\left|S_{v}\right|$ is the number of elements on $S_{v}$, and |S| is the number of elements on *S*.

In previous studies, the related DT algorithms have typically been applied to functions of the relevant areas of specialization, such as churn prediction [51], forecasting corporate credit ratings [52], object classification in autonomous driving [53], and the identification of a route selection strategy in classification [49]. Thus, it is an effective method for the selection of medical research.

#### 2.3.3. Random Forest and Its Practical Applications

RF is a novel and highly effective machine learning technique. It is a type of ensemble-learning approach [54] that combines predictors of trees such that trees are highly dependent on random vector values for independent samples with the same classification as those in decisional trees in a constructed forest. The basic concept of RF is to assemble multiple DTs, bag, and bootstrap [7], and identify the classification results with negative or positive information voted using individual decision trees. It is capable of handling large datasets and process a large number of input variables without first deleting variables. RF classifiers can provide two measurements of randomness for a comprehensive view of a tree set: the first is for data, and the second is for features.

RF can be applied in diverse domains due to its features, and particularly in clinical diagnosis [55]. Thus, RF was used to processing the CVD problem in this study.

#### 2.3.4. Multilayer Perceptron and Its Applications

MLP works on a supervised neural network model that uses a simple perceptron learning algorithm and an original backpropagation for training. MLP is adopted in most series forecasting approaches [56]. It usually has three types of layers of neurons: the input layer (i.e., for some source nodes), hidden layers (i.e., for computational nodes), and the output layer, which highlights the learning performance and the MLP classifier [57]. Some hidden nodes possess the ability of MLP learning classifiers with a minimum of hidden nodes; hence, the network is the most recommended and selected.

**Figure 1:**
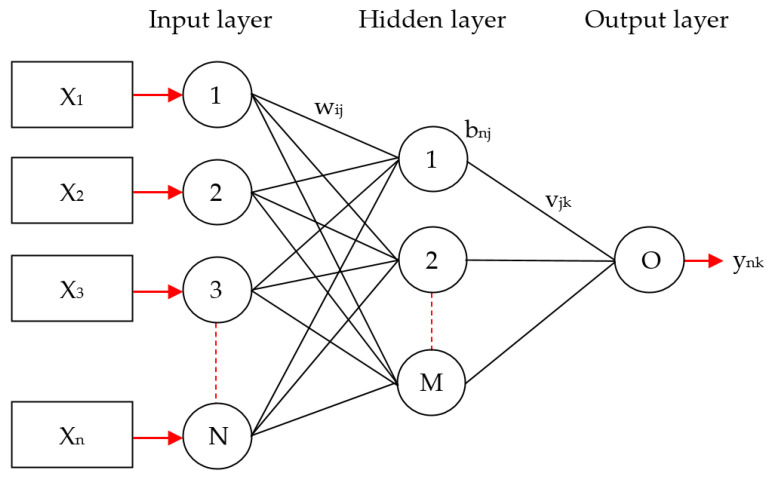
Schematic diagram for multilayer perceptron (MLP).

Regarding the layers for the MLP, each layer has a plurality of processing units, and each unit has subsequent layers in unit-weighted links. An MLP with inputs from a plurality of nonlinear variables to an output [58] is depicted in Figure 1.

If an input vector of an input layer is acquired from an output value b*_nj_* of hidden nodes, the following equation is defined:

(4) $$b_{nj}=\varphi (\theta_{M}+\overset{n}{\underset{i=1}{\sum }}w_{ij}\cdot x_{i}),$$

where φ refers to an activation function from the hidden nodes, $\theta_{M}$ represents a threshold from the hidden nodes, $w_{ij}$ refers to a connection weight between the hidden layers and the input layers, and $x_{i}$ is used as the input vector from these input nodes. The only output from these output nodes is acquired from the nodes of a hidden layer in a similar way:

(5) $$y_{nk}=\varphi (\theta_{k}+\overset{m}{\underset{i=1}{\sum }}v_{jk}\cdot b_{nj}),$$

The activation function used is sigmoid and defined as:

(6) $$S\left(t\right)=\frac{1}{1+e^{-t}}.$$

The training of an MLP network is supervised. MLP can use the gradient descent algorithm [59] for training or use the conjugated gradient algorithm with a nonlinear optimal algorithm. It is possible to accelerate the convergence speed of the weights with respect to the gradient descent algorithm.

Due to its excellent prior performance in medical applications [60], MLP was also selected for comparative analysis of the proposed study model.

#### 2.3.5. Support Vector Machine and Its Industrial Applications

SVM, invented initially by Vapnik, is one of the most common machine learning approaches used, with excellent performance in various applications [61]. SVM is a supervised learning method [62] with the main functions of regression and classification; it separates linearly separable input data into preset classes using the hyperplanes from an n-dimensional space. Using an optimal separated hyperplane maximizes the margins between data points that are nearest to each other within the different known classes. SVM was used in many prior studies [63,64,65] and has a good professional reputation due to its ability within a binary classification model.

Based on its strong positive reputation for medical research [66], SVM was thus selected and used for one of the proposed models in this study.

## 3. Proposed Method

To achieve the study goal, another key function of a classification technique is to classify an unknown category of given data objects corresponding to the known category for prediction purposes. The most challenging task of classification techniques is to learn how to select a suitable technique to improve the accuracy in the medical field. Previous studies of Framingham risk attributes have used statistical methods conducted with the SPSS software, in addition to the area under the curve (AUC) of the receiver operating characteristic (ROC) curve, sensitivity, and specificity, to show the prediction power for addressing CVD problems during the past 10 years [12]. However, they did not apply the Framingham risk score to intelligent machine learning classification techniques for identifying CVD and, in particular, for the extraction of knowledgeable rules. Thus, the above gaps should be bridged using well-defined methods of techniques used in medical research applications, which is the focus of this study. This study proposed using the Framingham risk score to filter attributes and then employing five classification techniques to build a hybrid model for identifying CVD. Two CVD datasets were compiled for the verification of the proposed model, and the performances of the Framingham risk score, Framingham attributes, and the full attributes in five classifiers were compared.

**Figure 2:**
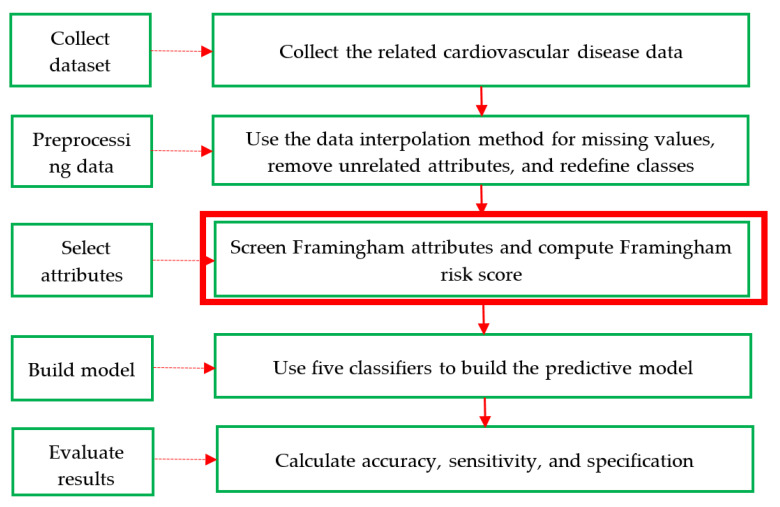
Research procedure of the proposed method.

The proposed method has five steps: compile the dataset → preprocess the data → select the attributes → build the model → evaluate the results. Figure 2 shows a flow diagram of the algorithm proposed in this method, and detailed information about the proposed algorithms is provided in the following.

- Step 1: Collect dataset. An adequate dataset is key in the identification of CVD; thus, this study compiled two datasets with CVD data. The first was compiled from a real prevention and treatment plan of CVD in Taiwan’s regional teaching hospital and had 20 attributes with 1190 records (including 551 men and 639 women). It is referred to as the CVD dataset in this study. The second dataset is a Framingham public dataset collected with an original 18 attributes and extracted from the University of Washington (http://courses.washington.edu/b513/datasets/datasets.php?class=513) via a uniform resource locator. It is referred to as the Framingham dataset.
- Step 2: Preprocessing data. In general, real data may have inconsistencies, gaps, errors, inaccuracies, impossible data combinations, and noise compared to the original dataset. Such data are not suitable for use by machine learning to discover hidden information/knowledge from large datasets. However, this step cannot remove these values. The average values of the data interpolation method are filled with missing values, and the dataset is then reformatted appropriately.

The first CVD dataset is employed to illustrate the proposed method. Based on a literature review, there are eight types of related CVDs, such as arrhythmia, cardiogenic shock, and diabetes mellitus. Twenty original attributes are reduced to 13 according to the expert opinion of physicians. Consequently, the 13 attributes include five physical exam attributes, seven blood test attributes, and one decision-attribute of the class with the eight CVD names noted above. There are two categories in the class, that is, Y (Yes): Have at least one CVD and N (No): None. These are listed in Table 5 below. Next, the second dataset has all of the attributes, including age, sex, serum cholesterol, DBP, SBP, Metropolitan relative weight, smoke, and CVDs (class), which is based on the expert recommendation of doctors. For further understanding of these datasets, their properties are shown in Table 6.

- Step 3: Select attributes. First, the eight attributes of the Framingham risk score are age, gender, DBP, SBP, LDL-C, HDL-C, fasting glucose, and smoking, which are named the Framingham attributes. Next, these factors are selected to calculate and transform their score values based on the conversion method of the Framingham risk score discussed in Section 2.2 [28] and shown in Table 2, Table 3 and Table 4 from the CVD dataset. Finally, all 13 attributes of the CVD dataset are selected and called the full attributes, which additionally include body mass index, waistline, red blood cells, and white blood cells.
- Step 4: Build a model. This step designs a hybrid model of five classifiers (i.e., RST, DT, RF, MLP, and SVM) with various attribute components, which include the Framingham score attributes, the Framingham attributes, and the full attributes, to highlight and differentiate the performance of the proposed method based on a commonly used 67–33% training–testing ratio. This step can be divided into five sub-steps and is executed using different software packages. The procedure of the five sub-steps is as follows: First, the selected attributes are used as input variables. Second, for the percentage-split data method, the two training–testing sub-datasets are formed using the common 2:1 ratio to achieve a good and reasonable result in practice. Thus, 67% of the data is used as a training sub-dataset, and the remaining 33% of the data is used as the testing sub-dataset. Third, all of the default parameters are defined to implement each of the above classifiers. Fourth, RST is applied using the rough set exploration system (RSES) [29], and DT, RF, MLP, and SVM are applied separately. Fifth, comprehensive knowledge-based rule sets are created using DT. For further details, the pseudo-code of the construction of the hybrid model is shown in Algorithm 1.

**Algorithm 1**: Pseudo Code of Building Hybrid Model**Input:** SA = selected attributes list, CD = collected data, RSES = rough set exploration system Use attributes from SA; Create 67% of data for training from CD; Create the other 33% of data for testing from CD; Define all the default parameters; Apply RST classifier by RSES; Apply DT, RF, MLP, and SVM classifiers, respectively; Create tree-based rules sets by DT; **Output:** knowledge(tree)-based rules sets

- Step 5: Evaluate the results. To evaluate machine learning, most researchers use three common metrics: accuracy, sensitivity, and specificity. These are defined in Table 7 of the confusion matrix and Equations (7)–(9). In Table 7, a true positive (TP) means the truth is positive, and the prediction is positive; a false positive (FP) means the truth is negative, but the prediction is positive; a false negative (FN) means the truth is positive, but the prediction is negative, and a true negative (TN) means the truth is negative, and the prediction is negative.

(7) $$accuracy=\frac{TP+TN}{TP+TN+FP+FN},$$

(8) $$sensitivity=\frac{TP}{TP+FN},$$

(9) $$specificity=\frac{TN}{TN+FP}.$$

## 4. Experiment Result and Comparisons

This section verifies the proposed method and its algorithms with two medical datasets and compares the listing models to further evaluate the classification performance.

### 4.1. Introduction and Preprocessing of the Two Medical Datasets

To further explore the experimental CVD dataset, Table 8 shows the descriptive statistics for the first CVD dataset using the chi-squared test to summarize and compare the baseline characteristics of the continuous attributes and the categorical attributes.

### 4.2. Experimental Results

The extracted entropy-based decision rules, core attributes, accuracy rate, sensitivity, and specificity were calculated from the two medical datasets after the experiments.

#### 4.2.1. The CVD Dataset

After data preprocessing and attribute selection, the Framingham attributes and full attributes were identified, and the five different classifiers were used and compared with various evaluation standards for the overall accuracy rate using a training–testing ratio of 67–33% to measure the performance of the proposed method.

**Figure 3:**
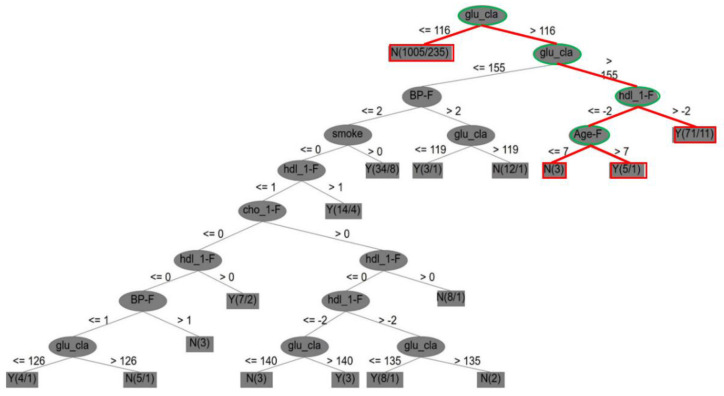
Entropy-based rule results of the cardiovascular disease (CVD) dataset in a visualized tree structure.

Consequently, the visualized tree structure of if–then–else control statements in the DT model was used to identify future CVD issues. Figure 3 lists the entropy-based rule results of the CVD dataset in a visualized tree structure. As shown in Figure 3, one case is exemplified and highlighted in red and green, and one key result of the core attributes is defined.

(1) First, it is indicated that, if the score measurement of glu_cla (i.e., diabetes) is higher than 116, glu_cla is also higher than 155, else if glu_cla is less than 116, there is no CVD case; if the score measurement of hdl_1-F (i.e., Framingham high-density cholesterol) is higher than −2, then there is a CVD case; else if Age-F (score of Framingham age) is higher than 7, there is a CVD case, but if it is less than or equal to 7, there is no CVD case.
(2) Second, the core attributes were obtained by dynamic reduction through discrete tables, and seven core attributes for Framingham risks were identified, including sex (i.e., gender), age_F (i.e., age), cho_1-F, hdl_1-F, BP_F, glu_cla, and smoke (i.e., smoking).

Accordingly, Table 9 shows the comparative results of various evaluation standards on the Framingham score attributes, the Framingham attributes, and the full attributes of the CVD dataset with five classifiers. In Table 9, it is clear that SVM outperforms the other four classifiers in terms of accuracy (99.67%), sensitivity (99.93%), and specificity (99.71%) for the three aspects of the Framingham score attributes, the Framingham attributes, and the full attributes. This implies that SVM is more suitable for identifying and addressing CVD than the other methods examined in this study.

#### 4.2.2. The Framingham Dataset

The second Framingham dataset with 18 original attributes was also considered. Similarly, after the preprocessing and identification of the Framingham attributes and the full attributes, this dataset was used with the five different classifiers to assess the evaluation performance of the proposed method in terms of average accuracy rate for 10 repetitions, also using a training–testing ratio of 67–33%.

**Figure 4:**
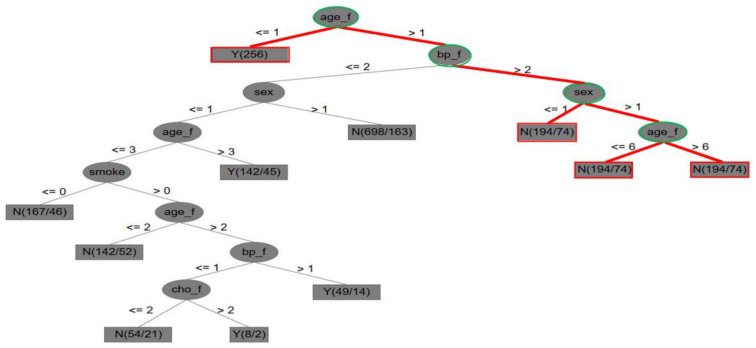
Entropy-based rule results of the Framingham dataset in a visualized tree structure.

DT was also used to visualize the tree structure of if–then–else control statements for identifying CVD. Figure 4 shows the entropy-based rule results of the Framingham dataset in a visualized tree structure. In Figure 4, two key points can be determined. First, a case from the tree-like structure is highlighted in red and green. Next, key attributes are accordingly identified and determined.

(1) In Figure 4, it is indicated that, if the score measurement of the Framingham attribute of age (age_f) is higher than 1, else if age is less than 1, there is a CVD case; if the score measurement of the Framingham attribute of blood pressure (bp_f) is higher than 2, and the sex (gender) score is higher than 1, then the age (age_f) score is higher than 6, and there is a CVD case; else if the score of Framingham age is less than or equal to 6, then there is no CVD case.
(2) Five key attributes were also obtained by dynamic reduction through discrete tables: sex, age_f (i.e., age), cho_f, bp_f, and smoke (i.e., smoking).

Table 10 lists the analytical results for the Framingham attributes and the full attributes in the Framingham dataset after the experiments. In Table 10, the five classifiers are compared with the three evaluation standards in terms of accuracy, sensitivity, and specification. It is clear that the RS method has the highest performance in terms of accuracy (85.11%), sensitivity (86.06%), and specificity (85.19%) in all Framingham score attributes, the Framingham attributes, and the full attributes for the Framingham dataset. This information implies that the rough set theory is more suitable for identifying CVD in the Framingham dataset than the other four classifiers examined in this study.

### 4.3. Findings

Three key findings and management implications follow from the experimental results:

(1) The advantage of Framingham attributes with classification techniques: Although previous studies have used Framingham attributes and statistical methods to process CVD, they did not use a hybrid model to integrate Framingham score attributes and the five noted classification techniques for the identification of CVD or differentiation of various classifier performances. This study closes these gaps, and the proposed method not only aids understanding of comprehensive CVD tree-like entropy-based rules but also helps to prevent or even solve CVD problems. In addition, the score use of Framingham attributes can be an effective clinical reference for doctors and health care workers to improve the identification of CVD. Thus, a key specificity and novelty in this study are that the Framingham risk attribute scores can be calculated and used to produce entropy-based decision rules. This has not been undertaken in previous research based on our limited literature review.
(2) The identification of key attributes for CVD: The key attributes of the original Framingham risk data were identified by Dr. Rupert Payne, University of Edinburgh, and were uniformly compared with those of the first and second medical datasets in the experiments of this study. Table 11 shows their comparative results from the following three perspectives for the CVD issue: (a) Table 11 shows that the key attributes of the Framingham risk, the CVD dataset, and the Framingham dataset are in the order of 9, 7, and 5, respectively, for identifying CVD. It was found that the number of Framingham key attributes significantly and positively affects the overall classification accuracy, which can be proven from the accuracy rates in Table 9 and Table 10. (b) The two datasets are missing key attributes, resulting in poor classification performance. If sufficient key attributes are addressed in the future, overall accuracy can improve. (c) The importance of the key attributes for identifying CVD is listed in a top-down order, which may provide helpful information.
(3) The technical implications of the classifiers used: Based on the experimental results of the two medical datasets, no classifier or model is suitable for all practical datasets used in different applications. Thus, an appropriate classifier must be first found and defined to address specific data in the machine learning community.
(4) The management implications of healthcare issues: Regarding management implications, a set of standards was provided from the entropy-based decision rules of a tree-like structure (e.g., Figure 3 and Figure 4) to help prevent or solve CVD problems in advance. (a) First, this can indirectly remind CVD patients how to self-manage. (b) Second, the experimental results allow doctors to provide patients with managerial suggestions, such as changes in eating habits, self-measurement and self-control of blood pressure, cessation of smoking, and increased exercise. (c) Finally, the Framingham risk attributes used to identify CVD with the classification techniques listed in this study can be regarded as an effective prediction model for processing CVD. The analytical results can be stored for doctors and health personnel as clinical references.

## 5. Conclusions

This study proposes a hybrid method to integrate and model Framingham risk attributes and five novel classification techniques—RST, DT, RF, MLP, and SVM—for the identification of key attributes that influence CVD and to highlight preventive practices in healthcare services. The study’s contribution consists of calculating the score of Framingham risk attributes and identifying CVD using a suitable classifier for different datasets in hybrid medical applications. This contribution differs from those of previous studies [67]. For verification using the three evaluation criteria (accuracy, sensitivity, and specificity), 1190 instances in the CVD dataset available from Taiwan’s regional teaching hospital and 2019 examples from the public Framingham dataset were used. SVM showed the best performance in terms of accuracy (99.67%), sensitivity (99.93%), and specificity (99.71%) in all of the F-attributes in the CVD dataset, and RS showed the best performance in the accuracy (85.11%), sensitivity (86.06%), and specificity (85.19%) in most of the F-attributes for the Framingham dataset. Consequently, three main points can be made regarding the contribution, specificity, and novelty of this study: (1) Regarding its contribution, this study supports meaningful entropy-based knowledgeable rules for visualizing a tree structure and differentiates the classifiers from the two datasets, resulting in three useful research findings and three helpful management implications for subsequent medical research and other interested parties. (2) Regarding its novelty, the study results provide novel evidence that indicates no classifier or model is suitable for all practical datasets of medical applications. Thus, finding an appropriate classifier to address specific medical data is highly important. Furthermore, this study is the first to calculate and identify the use of key Framingham risk attribute scores, integrated with the five classification techniques noted above and with the DT technique, to produce entropy-based decision rules of knowledge sets. This has not been achieved in previous studies. (3) Regarding the specificity of the study, the knowledgeable rule sets created by DT provide reasonable solutions to simplifying the processes of preventive medicine by standardizing the formats and codes used in medical data to address CVD problems. The specificity of these rules is thus significant compared to those of past research.

Five conclusive and important directions are indicated by the analytical results of the experiments using the two medical datasets:

(1) According to the empirical results, the CVD and Framingham datasets were processed into useful entropy-based tree information/knowledge by applying five classifiers to knowledge discovery in the databases. Furthermore, machine learning tools were found to be useful in medical applications with the integration of Framingham risk attributes. Regarding the experimental results, the support vector machine method showed a better performance using Framingham score attributes and five classifier techniques in terms of accuracy, sensitivity, and specificity in the CVD dataset. However, the rough set method outperformed the other classifiers in the Framingham dataset. In addition, through the entropy-based decision rule’s visualization of trees, the if–then–else control statement provides an understanding of how all decision items with a class status of “Y” or “N” (i.e., Yes or No) are identified in the two medical datasets to address CVD problems.
(2) Based on the literature review, machine learning and knowledge discovery using medical databases have attracted a significant amount of interest from academia and other fields. Given this interest in CVD applications, this study provides insights into data-mining techniques of machine learning and statistical methods with industry databases.
(3) This study concerns practical CVD applications and is a trial of machine learning techniques. It involves the extraction of helpful entropy-based decision rules, the anticipation of decision-making challenges for real-life applications of knowledge discovery, understanding of current results and insights, and exploration of future research directions.
(4) In Taiwan, the number of CVD patients has increased during the past 10 years due to changes in lifestyle and eating habits. Furthermore, CVD, cerebrovascular disease, diabetes, and hypertension were ranked second, third, fifth, and tenth among the top causes of death. It is clear that CVD is a serious issue; thus, early diagnosis and prevention strategies of CVD are important for healthcare and to decrease the use of medical resources.
(5) Several issues can be examined in subsequent research: (a) to further evaluate the proposed method, more attributes of Framingham risk can be collected to better predict CVD; (b) more classification techniques can be used and measured to predict CVD; (c) an alternative to the proposed method can be constructed with a variety of evaluation standards; and (d) more knowledge-based decision rules, which are not based on a variation of DT entropy, such as RSES of the rough set theory, can be provided for medical applications.

## Figures and Tables

**Table 1:** Top five causes of death in 2015 in Taiwan.

Rank	Origins of Death	Mortality
1	Malignant neoplasm	46,829
2	Cardiovascular disease	19,202
3	Cerebrovascular disease	11,169
4	Pneumonia	10,761
5	Diabetes mellitus	9530

**Table 2:** Conversion of Framingham risk score for age and cholesterol with gender differences.

Age			Cholesterol		
Years	Man	Woman	(mg/dL)	Man	Woman
30–34	−1	−9	<160	−3	−2
35–39	0	−4	160–199	0	0
40–44	1	0	200–239	1	1
45–49	2	3	240–279	2	1
50–54	3	6	≥280	3	3
55–59	4	7			
60–64	5	8			
65–69	6	8			
70–74	7	8			

**Table 3:** Conversion of Framingham risk score for high-density lipoprotein cholesterol (HDL-C) and blood pressure with gender differences.

HDL-C			Blood Pressure		
(mg/dL)	Man	Woman	Systolic/Diastolic (mmHg)	Man	Woman
<35	2	5	<120/80	0	−3
35–44	1	2	120–129/80–84	0	0
45–49	0	1	130–139/85–89	1	0
50–59	0	0	140–159/90–99	2	2
≥60	−2	−3	≥160/100	3	3

**Table 4:** Conversion of Framingham risk score for diabetes and smoking with gender differences.

Framingham Risk	Diabetes		Smoking	
	Man	Woman	Man	Woman
Yes	2	4	2	2
No	0	0	0	0

**Table 5:** The coding information for cardiovascular disease (CVD) dataset attributes.

Physical Exam		Blood Test		Class	
Attribute	Code	Attribute	Code	CVD Name	Code
Age	Age	Cholesterol	cho_1	Arrhythmia	arr
Body mass index	bmi_1	Diastolic pressure	dp_1a	Cardiogenic shock	car
Gender	sex	High-density cholesterol	hdl_1	Diabetes mellitus	dm
Smoking habit	smoke	Fast glucose	glu_c1a	High uric acid	uric
Waistline	wai_1	Red blood cells	rbc_1	Hyperlipidemia	lip
		Systolic pressure	sp_1a	Myocardial infarction	mi
		White blood cells	wbc_1	Narrow heart disease	hd
				Peripheral vein disease	pns

**Table 6:** Properties of the two medical datasets used.

Characteristic	CVD Dataset	Framingham Dataset
No. of instances	1190	2019
Class	Cardiovascular (Y: 358) Non-cardiovascular (N: 832)	Cardiovascular (Y: 983) Non-cardiovascular (N: 1036)
Total attributes	20	8
Full attributes	13	8
Framingham score attributes	8	6
Framingham attributes	8	7

Note: all the above attributes include one class attribute inside.

**Table 7:** Confusion matrix for classification.

		Predicted Class	
		Positive	Negative
**Actual Class**	Positive	True-positive	False-negative
	Negative	False-positive	True-negative

**Table 8:** Descriptive statistics of the CVD dataset.

Feature	Value	Number	Ratio (%)	*p*-Value	Note
Sex	Man	551	46	0.0743	Nominal
	Woman	639	54		
Age	40–44	19	2	0.0021 **	Numeric
	45–49	160	13		
	50–54	190	16		
	55–59	205	17		
	60–64	189	16		
	65–69	158	13		
	70–74	269	23		
Cholesterol	<160	189	16	0.3806	Numeric
	160–199	468	39		
	200–239	369	31		
	240–279	130	11		
	>=280	34	3		
HDL-cholesterol	<35	84	7	0.0121 *	Numeric
	35–44	364	30		
	45–49	222	19		
	50–59	306	26		
	>=60	214	18		
Systolic blood pressure	<120	330	28	0.0436 *	Numeric
	120–129	254	21		
	130–139	244	21		
	140–159	287	24		
	>=160	75	6		
Diastolic blood pressure	<80	451	38	0.9508	Numeric
	80–84	343	29		
	85–89	62	5		
	90–99	257	22		
	>=100	77	6		
Diabetes mellitus	No	1056	89	<0.001 ***	Nominal
	Yes	134	11		
Smoking	No	846	71	<0.001 ***	Nominal
	Yes	344	29		

Note: the “*”, “**”, and “***” refer to the significance threshold of *p*-value at 0.05, 0.01, and 0.001, respectively.

**Table 9:** The comparative results of various evaluation standards in the CVD dataset.

Evaluation Indicator		RS (%)	DT (%)	RF (%)	MLP (%)	SVM (%)
Accuracy	F score	80.81 (2.25)	82.49 (1.32)	79.87 (1.53)	72.54 (1.48)	91.23 (0.92)
	F attributes	75.75 (1.59)	83.00 (2.03)	81.95 (1.61)	72.54 (1.57)	99.67 (0.17)
	Full attributes	74.04 (4.19)	79.07 (2.34)	81.74 (0.65)	70.12 (3.54)	99.14 (0.11)
Sensitivity	F score	15.93 (9.46)	91.26 (3.02)	85.40 (2.29)	90.57 (3.52)	94.47 (0.89)
	F attributes	26.18 (6.92)	91.30 (2.57)	90.35 (1.96)	92.54 (3.51)	99.93 (0.20)
	Full attributes	21.16 (5.40)	87.29 (3.97)	91.96 (1.73)	85.95 (7.62)	99.10 (0.21)
Specificity	F score	95.07 (4.19)	71.15 (5.62)	66.24 (2.72)	69.36 (5.64)	87.99 (2.89)
	F attributes	91.31 (4.29)	72.85 (6.09)	70.81 (4.61)	73.93 (4.49)	99.71 (0.11)
	Full attributes	91.92 (4.09)	69.97 (5.26)	71.91 (3.77)	66.67 (8.65)	99.01 (0.41)

Note: each value and standard deviation (in brackets) is the average value of 10 repetitions. F score denotes the Framingham score attributes, and F attributes represents the Framingham attributes.

**Table 10:** The analytical results of the Framingham attributes and the full attributes for the Framingham dataset.

Evaluation Indicator		RS (%)	DT (%)	RF (%)	MLP (%)	SVM (%)
Accuracy	F Score	75.63 (0.9)	74.34 (1.53)	72.23 (1.44)	74.91 (1.38)	74.19 (1.35)
	F attributes	85.11 (2.27)	72.59 (1.21)	71.37 (1.06)	73.93 (2.93)	49.70 (0.88)
	Full attributes	76.79 (1.12)	72.35 (1.37)	71.73 (1.29)	73.61 (1.75)	63.33 (1.54)
Sensitivity	F Score	64.87 (3.18)	83.77 (2.81)	80.18 (1.92)	84.53 (3.23)	80.32 (3.39)
	F attributes	86.06 (3.07)	65.16 (5.29)	66.05 (2.23)	69.88 (7.07)	11.16 (1.79)
	Full attributes	77.74 (4.58)	82.31 (3.08)	77.31 (2.55)	82.19 (8.22)	61.29 (2.88)
Specificity	F Score	85.19 (2.76)	35.59 (2.45)	36.15 (2.87)	35.23 (3.69)	32.26 (3.15)
	F attributes	83.38 (4.61)	20.36 (5.07)	23.57 (1.27)	22.23 (10.25)	13.75 (2.21)
	Full attributes	74.80 (5.25)	38.15 (4.00)	34.15 (2.69)	35.44 (7.96)	34.51 (1.35)

Note: all terms are defined in Table 9.

**Table 11:** Key attributes of comparison for CVD issues.

Framingham Risk (9 ^1^)	CVD Dataset (7)	Framingham Dataset (5)
Family history of premature	Sex	Age
Sex	Diabetes	Sex
Age	Blood pressure	Blood pressure
Blood pressure	HDL-cholesterol	Smoking
Smoking	Smoking	Cholesterol
HDL-cholesterol	Age	
Diabetes	Cholesterol	
Cholesterol		
Left ventricular hypertrophy		

Note: ^1^ the values in the brackets refer to the total number of key attributes in the corresponding dataset.
